# CUPID COVID-19: emergency department attendance by paediatric patients during COVID-19 - project protocol

**DOI:** 10.12688/hrbopenres.13066.2

**Published:** 2020-08-10

**Authors:** Thérèse McDonnell, Eilish McAuliffe, Michael Barrett, Ciara Conlon, Fergal Cummins, Conor Deasy, Conor Hensey, Ciara Martin, Emma Nicholson

**Affiliations:** 1 IRIS Centre, UCD School of Nursing, Midwifery and Health Systems, University College Dublin, Dublin, Ireland; 2 Children's Health Ireland at Crumlin, Dublin, Ireland; 3 Women's and Children's Health, School of Medicine, University College Dublin, Dublin, Ireland; 4 National Children's Research Centre, Dublin, Ireland; 5 Limerick University Hospital, Limerick, Ireland; 6 Cork University Hospital, Cork, Ireland; 7 Children's Health Ireland at Temple St, Dublin, Ireland; 8 Children's Health Ireland at Tallaght, Dublin, Ireland

**Keywords:** Paediatric, COVID-19, non-COVID illness, emergency care, delayed attendance, healthcare avoidance

## Abstract

**Background:** Measures introduced to delay the spread of COVID-19 may result in avoidance of emergency departments (EDs) for non-COVID related illness. Clinicians and medical representative bodies such as the Irish Association for Emergency Medicine (IAEM) have expressed concern that some patients may not seek timely urgent medical intervention. Evidence from previous epidemics found that hospital avoidance during outbreaks of MERS and SARS was common. While ED attendance returned to normal following SARS and MERS, both outbreaks lasted 2-3 months. As the COVID-19 pandemic is forecast to extend into 2021, little is known about the impact COVID-19 will have on paediatric attendance at EDs as the pandemic evolves.

**Aims**: This project aims to assess the impact of COVID-19 on paediatric emergency healthcare utilisation, to understand how the health seeking behaviour of parents may have altered due to the pandemic, and to identify how any barriers to accessing care can be removed.

**Methods**: Administrative data records from five EDs across Ireland and one Urgent Care Centre will be analysed to identify temporal trends in attendances for emergency care. Qualitative inquiry will be utilised to capture the experience of staff providing emergency healthcare to paediatric patients during COVID-19, and their feedback on identified trends will inform the interpretation of findings. A cross-sectional survey of parents will capture experiences, concerns and decision-making on accessing healthcare for their children during the pandemic.

**Results and Conclusion: **This information will help decision makers respond rapidly to meet the clinical needs of paediatric patients as the circumstances of the pandemic unfold and reduce the disruption to normal paediatric ED services during the onset of COVID-19. As the health of a child can deteriorate more rapidly than that of an adult, any delay in seeking care for an acutely ill child may have serious consequences.

## Introduction

The repurposing of health system resources to tackle the challenge of the COVID-19 pandemic, together with an apparent reluctance by some parents to access healthcare for their children during the pandemic, has led to a decrease in emergency department (ED) attendance by paediatric patients ^1^. Measures introduced to delay the spread of COVID-19 may result in avoidance of EDs for non-COVID related illness, with clinicians and medical representative bodies, such as the Irish Association for Emergency Medicine (IAEM), expressing concern that some patients may not seek timely medical attention for conditions which require urgent medical intervention. A delay in seeking healthcare, particularly for children with chronic complex conditions ^2^, may result in further deterioration in an illness and ultimately lead to hospital admissions which could have been avoided with timely access to healthcare ^1^. Avoiding unnecessary admissions to hospital is critical at a time when resources in the health system are severely limited.

Recent evidence on delayed paediatric ED presentations emerging from Italy, which experienced one of the deadliest outbreaks of COVID-19, underscores the risks associated with not seeking timely healthcare. Of 12 delayed presentations documented in a single week in March 2020, one child died en route to hospital by ambulance and six children were admitted to the ICU on arrival, of which four later died ^1^. These children had persistent and severe symptoms including polydipsia (excessive thirst), polyuria (excessive production of urine), high temperature, respiratory distress, convulsions and vomiting, and were diagnosed on arrival at hospital with life threatening conditions, such as type 1 diabetes, acute onset leukaemia, bacterial pneumonia, and severe hypoglycaemia. In each case, parents delayed seeking hospital care due to fear of contracting COVID-19. While five of these families did contact health services before accessing care at the ED, the healthcare provider was either unavailable due to the pandemic, or they were advised not to attend hospital due to the risk of contracting COVID-19. In England, ED attendances dropped by 25% in the week following the announcement of the lockdown on 23 ^rd^ March 2020 ^3^. In Ireland, visits fell by 38.3% among those aged under 10 and by 50% for those aged between 10–19 in March 2020 compared to March 2019, the period before the country entered the *Mitigation* phase ^4^. An Irish single-site study presented findings relating to presenting conditions for the two-month period from March to April, reporting a drop in total presentations for digestive disorders (56.5%), respiratory conditions (50%) and injury and poisoning presentations (44.7%) when compared to 2019 ^5^. 

Evidence from previous epidemics, such as the H1N1 and MERS outbreaks in Hong Kong and South Korea, found that hospital avoidance during such outbreaks was common ^6– 8^. Moreover, during the 2003 outbreak of SARS in Toronto, the decrease in high-acuity attendances at the ED was 3-times higher than low-acuity, which suggests that access to healthcare for critically ill patients was seriously affected ^9^. Evidence on paediatric patients at a mixed adult/paediatric hospital in South Korea found that, although attendance fell by 42.3% during the MERS epidemic, visits for trauma and the rate of admissions increased ^8^. While ED attendance returned to normal following SARS and MERS, both outbreaks lasted 2–3 months. The impact of an extended pandemic with stringent lockdown conditions is likely to be more severe.

School closures and the cancellation of sporting activities, social distancing and living in “lock-down” will lead to a reduction in ED attendances by children for trauma and acute infections ^1^. Also, the incidence of COVID-19 among children is low ^10^. Fewer than 1.5% of COVID-19 cases in Italy related to children, only 11% of whom were hospitalised, and no admittances to the Intensive Care Unit or fatalities were recorded ^1^. However, the prevalence of chronic conditions such as type 1 diabetes and cancer are not impacted by the pandemic and a decrease in presentations of this nature is cause for concern.

This project aims to determine if children continue to receive timely access to emergency healthcare while COVID-19 presents a challenge to the health system and restrictions such as social distancing remain in place. As the pandemic unfolds, assessment of changes in ED attendance and service provision will provide pertinent information to help identify unmet need and those at risk. A survey will provide insight into the experience of parents accessing emergency healthcare for their children during the pandemic and establish any avoidance or delay in seeking care. This study will also gather data from staff providing emergency healthcare to paediatric patients on their experiences during COVID-19, and their feedback on identified trends will inform the interpretation of findings.

## Research design and methodological approach

The research design for this project consists of three work packages. Leveraging on the work of CUPID (children’s unscheduled primary and emergency care Ireland - decision-making and trends), an ongoing HRB-funded project ^11^, all three work packages will be carried out simultaneously in collaboration with Children’s Health Ireland (CHI) at Crumlin, Temple St and Tallaght, Connolly Urgent Care Centre, Cork University Hospital, and Limerick University Hospital. This study combines a multi-method approach, including statistical analysis of temporal trends in attendance for emergency care, literature review, survey and qualitative inquiry through interview. Each work package will further the insights gained in other streams of the project to form a holistic picture of paediatric ED attendance throughout the pandemic.

The study will take place from 11 ^th^ May 2020 to 10 ^th^ May 2021.

### Work Package 1: temporal trends in paediatric attendance at EDs

***Methods.*** Work Package 1 will involve the extraction of anonymised retrospective patient visit records for children aged 15 ^1^ and under from the ED management systems of three paediatric, two mixed adult/ paediatric EDs, and one Urgent Care Centre. Scheduled attendance at EDs will be excluded from this analysis. Existing report formats designed by staff at each of the participating hospitals to extract data for CUPID ^11^ will be used to extract attendance records covering the period from 1 July 2018 to 30 April 2020, augmenting an existing database of visits compiled for CUPID. Thereafter, monthly files of attendance will be extracted up to 31 March 2021, to ensure patterns in attendance are assessed in the year following the identification of the first COVID-19 case in Ireland and the subsequent introduction of containment measures.

The variables to be extracted, as detailed in Table 1, include an anonymised unique patient identifier, which allows visits by individual patients to be connected at each hospital, though not across hospitals. Variation in how data is captured means not all variables are available from each participating hospital. For example, although each hospital records the reason for attendance, categorisation between hospitals varies substantially. The International Classification of Disease (ICD) coding of diagnosis is available for the CHI hospitals ^12^, and this will facilitate a granular analysis of changes in attendance by illness.

**Table 1.** Variables to be extracted from emergency department (ED) systems.

Patient static record	Visit data
Unique patient identifier (randomly generated and anonymised) Year and month of birth Gender	ED visit date and time Date and time of leaving ED Indicator of return visit General Medical Services (GMS) status (indicator of medical card status) Mode of arrival Source of referral (self-referral, referral from GP or Co-op GP, other) Triage score Presenting complaint Diagnosis (where available) Discharge outcome (home, admission, referral, transfer to intensive care unit, other transfer)

Each participating institution will also be asked to complete a survey detailing structural and process adaptions, as well as outlining changes to the local healthcare pathways triggered by the COVID-19 outbreak that might affect the number and type of presentations to the ED.

***Data analysis.*** Paediatric attendance at the ED throughout the duration of the pandemic will be compared graphically and statistically to attendance patterns over prior years using STATA 16. Preliminary analysis will involve the graphing of trends in paediatric attendance at the ED from the onset of COVID-19, with a particular focus on attendance following significant events in the course of the pandemic in Ireland, such as the HSE’s first meeting on COVID-19 preparedness, the Health Service Executive (HSE)’s first notification to hospitals to prepare for COVID-19, the identification of the first case of COVID-19 in the island of Ireland on 28 ^th^ February 2020, the first school closure on 1 ^st^ March, nationwide school closures on 12 ^th^ March, mandatory stay-at-home measures introduced from 28 ^th^ March, and the pandemic trends (recognition, acceleration, peak and deceleration) of COVID as described by the Centers for Disease Control (CDC) ^14^. Attendances on a daily, weekly and monthly basis will be compared with the same week and month in prior years, using moving averages where appropriate to account for daily volatility.

Descriptive statistics will be used to analyse ED attendance in each study period compared with prior periods, and to describe the pattern of ED attendance during the pandemic by subgroups. Variation in the mean and standard deviation of daily attendance throughout the public health stages will be assessed. Negative binomial regression will be used to compare relative changes in ED use between times and to compare variability of referrals, length of time in the ED, admissions and triage by factors such as age and, where data permits, presenting complaint and diagnosis. In particular, changes in presentations for illnesses considered to be high-risk in the context of late presentation, such as sepsis and diabetes, and changes in the level and nature of admissions to the intensive care unit (ICU) from the ED, will be identified.

Statistical and graphical analysis will be conducted at a total, regional and hospital level to determine the variability in the effect of the pandemic. Detailed mapping of diagnostic coding will allow the identification of vulnerable cohorts, in particular those presenting with chronic complex conditions, that may be avoiding EDs following the introduction of these measures. Mental health presentations will be assessed as living conditions alter in response to the prevalence of COVID-19. Correlations between attendance and county-level incidence of COVID-19 will also be assessed. This analysis will be updated throughout the duration of the project to assess how the nature of paediatric ED attendance evolves over the course of the pandemic in response to changes in the severity of COVID-19 and the living conditions imposed by the Irish Government.

### Work Package 2: semi-structured interviews with frontline staff

***Methods.*** Semi-structured interviews will be conducted with consultants and other front-line staff (n = 15) at each of the five participating hospitals and Connolly Urgent Care Centre. Staff from the ED and departments affected by changes made to the ED in response to COVID-19 will be identified by study collaborators at each of the participating hospitals. Interviews will be conducted by a trained interviewer by telephone, will be recorded, and will follow an outline topic guide. Each interview will aim to capture the following:

- the respondent’s experience of attendances following the onset of COVID-19, non- or delayed attendance for non-COVID illness, in particular by patients with chronic complex conditions such as diabetes and cystic fibrosis, and any increased attendance of patients with COVID related and non-related symptoms;
- their experience of how the behaviour of parents attending with their children has altered, and if parents have shared their concerns about accessing the ED during the pandemic;
- how their department has responded to the pandemic and how they have found working under the prevailing conditions;
- their views on mechanisms that could be applied to reach and support vulnerable patients throughout the duration of the pandemic.

***Data analysis.*** Interviews will be transcribed and de-identified. Using NVivo software, thematic analysis ^15^ will be utilised to analyse the data from interviews with ED staff in order to identify common themes and interpret findings. This will harness the insights of ED staff to contextualise the findings of Work Package 1.

### Work Package 3: cross-sectional survey of parents

***Methods.*** A cross-sectional national survey of parents will be conducted to gather their experience of accessing healthcare for their child during the onset of COVID-19. The primary aim of this survey is to identify the extent of healthcare avoidance by parents of children aged under 16 during the pandemic and to identify the reasons behind decisions to avoid or delay accessing healthcare for their children. Building on the qualitative data from interviews conducted with parents as part of the CUPID project and a review of the literature on avoidance of healthcare during an epidemic, pandemic or major population health crisis ^1, 3, 6– 10, 16– 18^, the survey will capture participants’ (n = 1000) experience of utilising unscheduled paediatric healthcare (e.g. EDs, urgent care centres, injury units, GPs, tele-medicine and online helplines) during the outbreak of COVID-19 and establish any avoidance of healthcare services. Online survey software provided by Qualtrics ^™^ will be used to design and administer the survey, which will capture the following:

- demographic information to profile parents, including highest level of parental education, medical card status, number of children and gender;
- relevant health information for the respondent and their children (e.g. pre-existing conditions and/or chronic illness/disability);
- the respondent’s experience of COVID-19, in particular if they or a close contact have had COVID-19, the severity of the illness, and if they lost someone to the illness;
- the respondent’s level of stress (e.g. the stress subscale of DASS-21 ^19^) at the time of the survey;
- accessibility of emergency healthcare including the ED, GP and Local Injury Unit;
- Use and/or avoidance of unscheduled healthcare services for their children during the pandemic;
- Use and experience of remote consultations and monitoring for healthcare (e.g. on-line advice, telephone or video consultations, monitoring apps).

The survey will be conducted in early June 2020, as Ireland begins to emerge from the most restrictive period of public health measures. While utilisation and hesitancy of parents on accessing healthcare for their child will rely on recall, the short recall period and the severity of the living conditions of the lockdown should facilitate accurate recall. One limitation is that the stress levels of the respondent can only be accurately captured at the time of the survey, and may not be representative of stress levels during the lockdown period or at the time of decision making on accessing healthcare, if relevant.

Respondents will be drawn from an existing Qualtrics panel, must be living in Ireland and have at least one child aged under 16 living with them, and are required to give their consent before commencing the on-line survey. All data collected and provided to CUPID COVID-19 by Qualtrics will be anonymous.

***Data analysis.*** The results from the survey will provide valuable information regarding the experiences and behaviour of parents when accessing necessary healthcare for their child following the onset of COVID-19. Descriptive statistical analysis will assess the prevalence of healthcare avoidance by parents during the pandemic. Regression analysis will determine the association between key demographics, experiences and decision making by parents on accessing or delaying healthcare for their child during the pandemic. Sub-group analysis will identify cohorts that may be vulnerable to delaying access and the prevalence of specific reasons, beliefs or concerns that mediate this decision.

### Ethics

Ethical approval has been granted by the COVID-19 National Ethics Research Committee, a committee established by the Minister of Health to deliver an expedited review process for COVID-19 research, for each work package in the CUPID COVID-19 project. Due to the collection of non-personal and anonymous data, explicit consent is not required for the data collected in Work Package 1. In accordance with data protection regulations, the data will be anonymised at each site by relevant hospital staff before being transferred securely to the research team. No identifiable data will be included in the final consolidated dataset. Written consent will be obtained for the semi-structured interviews and on-line consent will be obtained from parents completing the survey.

### Data availability

The visit records extracted for use in this study will be fully anonymised prior to transfer from each participating site. All data will be consolidated in an anonymised master database and a supporting data dictionary will record definitions for each variable. Further ethical approval will be required should it be deemed appropriate to deposit this dataset with a Repository. Data associated with the survey of parents will be deposited with the Irish Social Science Data Archive (ISSDA) and the de-identified transcribed interviews with frontline staff will be made available from the Irish Qualitative Data Archive at Maynooth University.

### Dissemination

Results will be disseminated via regular briefing notes, publication in peer-reviewed journals, national and international conferences, and to relevant stakeholders and interest groups through the use of public forums and social media.

## Study status

This study commenced on 11 ^th^ May 2020.

## Discussion

The primary aim of this project is to explore if children continue to receive timely access to emergency healthcare throughout the duration of COVID-19. A delay in seeking healthcare, particularly for children with chronic complex conditions ^2^, may lead to an otherwise avoidable hospital admission and a serious escalation in ill health for the child. This protocol details the approach and methods that will be adopted in assessing the impact of COVID-19 on paediatric ED attendance. This paper describes the quantitative and qualitative methods that will be applied to the extraction of data and the evaluation of attendance, preferences and experiences relevant to the utilisation of emergency healthcare by children as the pandemic unfolds. Through collaboration with clinicians involved in the delivery of paediatric emergency healthcare and gaining an understanding of parent’s behaviour through the survey, this study will provide a context rich understanding of paediatric emergency healthcare during this health emergency. The findings will provide valuable information on parents’ views and decision-making on accessing healthcare for their children during this time and examine the relationship with demographics and identified stressors. By assessing the experience of remote medicine, it will also give some insight on parents preferred mechanisms for receiving support and medical care throughout the pandemic to ensure patients continue to seek necessary healthcare for the duration of COVID-19.

The key challenges associated with this study relate to data accessibility and quality. The absence of a comprehensive national patient database of emergency care attendances requires the collation of datasets from a sample of hospitals. Data collected for clinical and administrative purposes may have gaps and inaccuracies that need to be accounted for in the analysis of the data. The extent of data capture will also vary between hospitals, resulting in missing or incomplete data for some observations.

 A key feature of this study will be the rapid publication of findings. Initial findings from the analysis of attendances will be shared within eight weeks of the commencement of the project. Monthly updates will ensure stakeholders have timely statistics on the impact of COVID-19 on paediatric ED presentations. Results from both the survey of parents and interviews with front-line staff at each participating ED will be communicated rapidly following completion of Work Packages 2 and 3. Findings from this research will be presented by the research team at national and international conferences where possible, and published in peer-reviewed journals. Communication of research findings and updates targeted at parents, children and the general public will be through accessible mediums of lay summaries and social media, designed to reach a wide public audience. Peer reviewed publications will be made available as open access articles and advertised on public forums to reach as broad an audience as possible.

The findings from this research will be of international relevance as health systems and populations around the world respond to the COVID-19 pandemic. The rapid release of findings will provide clinicians and other decision makers both within Ireland and internationally with time-critical information. Health systems must evolve to meet the changing needs of populations and this research will provide valuable insights into the unprecedented effects of the COVID-19 pandemic on paediatric emergency healthcare, as the system responds rapidly to meet significant capacity and safety challenges.

### Strengths of this study

- This study will extract a large sample of paediatric patient records of visits to EDs to assess the impact of COVID-19 on the pattern of attendance and non-attendance.
- Leveraging the data extraction processes and analysis developed for CUPID will ensure results are communicated promptly, and monthly updates will allow the impact on paediatric ED attendance of COVID-19 and the restrictions introduced at a national level to be monitored over the duration of the pandemic.
- The factors that influence the decision making of parents accessing emergency healthcare for paediatric patients during the pandemic will be identified through a national survey of parents.
- The experiences of frontline staff responsible for the delivery of emergency healthcare to paediatric patients during COVID-19 will provide valuable context to the findings of both the data analysis and survey of parents.

## Data availability

No data is associated with this article.

## Notes

^1^ The agreed age for paediatric emergency department attendance in Ireland is 16, the eve of 16 ^th^ birthday ^13^.
