# Supplementary material for: CUPID COVID-19: emergency department attendance by paediatric patients during COVID-19 - project protocol
Source: HRB Open Res. 2020 Aug 10;3:37. Originally published 2020 Jun 9. [Version 2] doi: 10.12688/hrbopenres.13066.2 (PMC7327719; doi:10.12688/hrbopenres.13066.2)
Supplement: Peer review history

# Peer review: Reviewer response for version 1

**DOI:** 10.21956/hrbopenres.14165.r27534

**Published:** 2020-06-30

**Authors:** Aoife Brick, Conor Keegan

**Affiliations:** 1 Social Division, Economic and Social Research Institute, Dublin, Ireland; 2 Social Division, Economic and Social Research Institute, Dublin, Ireland

This study will examine the impact of the COVID-19 pandemic on paediatric emergency department attendances. The study protocol outlines a multi-method approach with three work packages which will employ both quantitative and qualitative techniques incorporating administrative and survey data. This protocol is well written and clearly sets out the strands of work to be undertaken.

 It is timely and will provide important new information which to date has not been available for Ireland. In particular, the analysis of presenting condition in work package one should be very informative.

 The results of the study will be of interest both nationally and internationally. Some comments are outlined below.

**Specific comments:**

1. The role of the GP is not discussed. Was it considered to include GPs in the frontline interviews in WP 2? Are patients presenting at ED sicker because they are foregoing attending their GP or were unable to access a timely GP appointment. This appears to be suggested in the Italian paper presented (page 3, para 2).
2. Page 3 para 3 (end): provides an analysis of changing patterns of ED attendance following COVID-19 onset in Ireland.
3. Page 3, para 6: In terms of WP1 a concern relates to the distribution of centres. Four located in Dublin and two in South-West. It is unclear how nationally generalisable the findings will be - have the authors considered this? Dublin was relatively hard hit by COVID-19 which may have impacted ED attendance differently to other parts of the country.
4. Page 4, para 4: Related to the previous point, it might be useful to incorporate some temporal measure of county-level (or more granular if available) case incidence and examine how it correlates with attendance at ED.
5. Page 4, para 4: the authors could be more explicit about the statistical methods they intend to use to compare attendance patterns over time. Do the data available lend themselves to anything other than descriptive statistics?
6. Page 4, para 5: the authors could be more explicit about what is meant by ‘vulnerable cohorts’. Is this solely diagnosis-based? How exactly will they be identified?
7. Page 5, para 2: STATA is listed as a software package for the quantitative analysis – if a counterpart has been identified for the qualitative work, it could be listed for consistency.
8. Page 5, WP 3: It is not clear whether the cross-sectional survey of parents will be nationally representative or whether it is aligned with centres from WP 1/2. Presuming it is nationally representative, care will need to be taken if integrating findings across work packages.

**Minor comments:**

- Page 1 – nursing misspelled in first affiliation.
- Abstract – methods: ‘…identify changes in attendances at emergency healthcare’. Suggest this is rephrased.
- Page 3, para 2: ‘excessive thrust’ should be ‘excessive thirst’.
- Page 3, para 6: ‘…including statistical analysis of temporal trends of attendance at emergency care…’ Again, consider rephrasing (e.g. temporal trends in attendances for emergency care?)

Is the study design appropriate for the research question?

Yes

Is the rationale for, and objectives of, the study clearly described?

Yes

Are sufficient details of the methods provided to allow replication by others?

Partly

Are the datasets clearly presented in a useable and accessible format?

Not applicable

Reviewer Expertise:

Both reviewers are health economists with substantial experience working on a broad range of issues relating to the Irish health system. Both have recently published specifically in the area of emergency department utilisation in Irish public hospitals.

We confirm that we have read this submission and believe that we have an appropriate level of expertise to confirm that it is of an acceptable scientific standard.

## References

[1] : COVID-19 and emergency department attendances in Irish public hospitals.2020; 10.26504/qec2020may_SA_lyons 10.26504/qec2020may_SA_lyons

# Response

**Published:** 2020-07-30

**Authors:** Therese McDonnell

**Affiliations:** University College Dublin, Ireland

Thank you both for reading this protocol and your feedback.  In response to your points:

- **The role of the GP is not discussed. Was it considered to include GPs in the frontline interviews in WP 2? Are patients presenting at ED sicker because they are foregoing attending their GP or were unable to access a timely GP appointment. This appears to be suggested in the Italian paper presented (page 3, para 2).**

Interviews with GPs would certainly provide valuable information.  This project is aligned to a larger project, CUPID, and interviews with GPs about their decision-making on referring paediatric patients to the ED form part of this aligned project.  Questions will be incorporated into these interviews to explore changes in access and referral practices by GPs to the ED during the pandemic.

 

- **Page 3 para 3 (end): provides an analysis of changing patterns of ED attendance following COVID-19 onset in Ireland.**

As two further studies on ED attendance during COVID-19 in Ireland have been published since this manuscript was first prepared, we have taken the opportunity to add these to this protocol due to their relevance.

“ *At present, no data exist to verify these findings in Ireland*” has been removed and the following amendment has been made to this paragraph:

*In Ireland, visits fell by 38.3% among those aged under 10 and by 50% for those aged between 10-19 in March 2020 compared to March 2019, the period before the country entered the Mitigation phase [18].* *An Irish single-site study presented findings relating to presenting conditions for the two-month period from March to April, reporting a drop in total presentations for digestive disorders (56.5%), respiratory conditions (50%) and injury and poisoning presentations (44.7%) when compared to 2019* *[19].*

1. Brick A, Walsh B, Keegan C, et al.: COVID-19 and emergency department attendances in public hospitals *. ESRI Special Article. *2020.
2. Dann L, Fitzsimons J, Gorman KM, et al.: Disappearing act: COVID-19 and paediatric emergency department attendances. *Archives of Disease in Childhood*. 2020.

 

- **Page 3, para 6: In terms of WP1 a concern relates to the distribution of centres. Four located in Dublin and two in South-West. It is unclear how nationally generalisable the findings will be - have the authors considered this? Dublin was relatively hard hit by COVID-19 which may have impacted ED attendance differently to other parts of the country.**

We have considered this. As work is currently underway with these six sites on an aligned project, the data extraction reports needed to prepare this analysis are already in place. As this project is part of the rapid response to COVID-19, it was felt the current collaborations would give a solid representation of the national picture. While generalisability cannot be assumed, the two regional hospitals do differ from the Dublin hospitals on a number of key points:

- CUH and LUH are both mixed adult/paediatric hospitals, serving a large community, while the Dublin EDs are dedicated paediatric facilities.
- The mixed urban/rural population served contrasts with that of the Dublin hospitals.
- Paediatric patients attending these hospitals are almost twice as likely to be referred by a GP than those attending the Dublin hospitals.
- They also offer a contrast in terms of COVID-19. As of 28 ^th^ July 2020, the current reported incidence of COVID-19 in Ireland is 0.54% of the population. The rate for the Dublin area is 0.81%, and 0.39% for the rest of the country. Cork and Limerick areas are both below the average for both the country and the area outside of Dublin at approximately 0.30%.
- **Page 4, para 4: Related to the previous point, it might be useful to incorporate some temporal measure of county-level (or more granular if available) case incidence and examine how it correlates with attendance at ED.**

Agreed. This has now been added to the Data Analysis section of Work Package 1.

- **Page 4, para 4: the authors could be more explicit about the statistical methods they intend to use to compare attendance patterns over time. Do the data available lend themselves to anything other than descriptive statistics?**

The preliminary analysis will track the mean and standard deviation of daily attendance throughout the public health stages.  This has now been specified in the commentary.

*Variation of the mean and standard deviation of daily attendance throughout the public health stages will be assessed. Negative binomial regression will be used to compare relative changes in ED use between times and to compare variability of referrals, length of time in the ED, admissions and triage by factors such as age and, where data permits, presenting complaint and diagnosis.*

- **Page 4, para 5: the authors could be more explicit about what is meant by ‘vulnerable cohorts’. Is this solely diagnosis-based? How exactly will they be identified?**

This is solely diagnosis-based. The protocol has been amended as follows:

*Detailed mapping of diagnostic coding will allow the identification of vulnerable cohorts, in particular those presenting with chronic complex conditions, that may be avoiding EDs following the introduction of these measures. Mental health presentations will be assessed as living conditions alter in response to the prevalence of COVID-19.*

- **Page 5, para 2: STATA is listed as a software package for the quantitative analysis – if a counterpart has been identified for the qualitative work, it could be listed for consistency.**

NVivo software will be used to analyse the data and the following sentence has been added to the ***Data analysis*** section of the work package 3 description:

*NVivo software will be used to analyse the data.*

 

- **Page 5, WP 3: It is not clear whether the cross-sectional survey of parents will be nationally representative or whether it is aligned with centres from WP 1/2. Presuming it is nationally representative, care will need to be taken if integrating findings across work packages.**

Noted. The nationally representative survey, conducted in early June, assesses parent’s hesitancy in accessing healthcare, not specifically care at an emergency department, for their children following the onset of the pandemic, since the closure of the school. As county of residence is captured in the survey, we will be able to assess if there is geographical variation in responses by parents.

“national” has been added to the description of the survey in the ***Methods*** section of work package 3.

**Minor comments:**

**Page 1 – nursing misspelled in first affiliation.**

**Abstract – methods: ‘…identify changes in attendances at emergency healthcare’. Suggest this is rephrased.**

**Page 3, para 2: ‘excessive thrust’ should be ‘excessive thirst’.**

**Page 3, para 6: ‘…including statistical analysis of temporal trends of attendance at emergency care…’ Again, consider rephrasing (e.g. temporal trends in attendances for emergency care?)**

All the above changes have been made.

# Peer review: Reviewer response for version 1

**DOI:** 10.21956/hrbopenres.14165.r27535

**Published:** 2020-06-30

**Authors:** Antonio Clavenna

**Affiliations:** 1 Department of Public Health, Laboratory for Mother and Child Health, Istituto di Ricerche Farmacologiche Mario Negri IRCCS, Milan, Italy

The decrease in emergency department attendances is a well described phenomenon occurring during epidemics.

 In part, it may reflect the fact that many accesses are for non urgent reasons and should be managed by the general practitioners, but it is also possible that attendances for urgency may be delayed for the fear of potential contagion, with risks for the health of the patient.

 In my opinion, the protocol of the study is well written. The study could provide helpful findings to identify interventions to guarantee emergency care to vulnerable children.

 I have one concern: it seems to me that WP3 activities (survey of parents) will be carried out after the peak of the epidemic (at least the "first wave"). I would like to suggest to discuss how the responses by parents could be influenced by the recall of a "previous" behaviour. Moreover, the current level of stress may not reflect the stress experienced during the lockdown.

Is the study design appropriate for the research question?

Yes

Is the rationale for, and objectives of, the study clearly described?

Yes

Are sufficient details of the methods provided to allow replication by others?

Partly

Are the datasets clearly presented in a useable and accessible format?

Not applicable

Reviewer Expertise:

Clinical pharmacology, epidemiology, drug utilization.

I confirm that I have read this submission and believe that I have an appropriate level of expertise to confirm that it is of an acceptable scientific standard.

# Response

**Published:** 2020-07-30

**Authors:** Therese McDonnell

**Affiliations:** University College Dublin, Ireland

Thank you for reviewing this protocol and for your comments. The survey was conducted in early June, as Ireland began to emerge from the most restrictive period of public health measures.  Some questions, as you correctly point out, did require recall by the parent. However, as the recall period was short and the living conditions of the lockdown so severe and unusual, we feel recall of the key questions on hesitancy and healthcare utilisation should be relatively accurate. The questions were adjusted to account for the timing of the survey.The level of stress captured is relevant to the time of the survey and may not capture stress levels during the lockdown.  This will be a limitation of this analysis.

The following has been added to the methods section of work package 3:

*The survey will be conducted in early June 2020, as Ireland begins to* *emerge from the most restrictive period of public health measures.  While utilisation and hesitancy of parents on accessing healthcare for their child will rely on recall, the short recall period and the severity of the living conditions of the lockdown should facilitate accurate recall. One limitation is that the stress levels of the respondent can only be accurately captured at the time of the survey, and may not be representative of stress levels during the lockdown period or at the time of decision making on accessing healthcare, if relevant.*
